# Assessing the Macroeconomic Determinants of International Tourist Arrival in India: An ARDL Bounds Testing Approach

**DOI:** 10.12688/f1000research.170280.1

**Published:** 2025-11-05

**Authors:** Ajit Kumar Singh, Idris Oyewale Oyelakin, Ravindra Singh Kushwah, Saurabh Bharti, Sandeep Paatlan, Muhammad Afnan Mahusain, Khalid Hamad Alturki, Anita Kumari Singh

**Affiliations:** 1 Chandigarh University, Sahibzada Ajit Singh Nagar, Punjab, India; 2 INTI International University, Nilai, Negeri Sembilan, Malaysia; 3 University of Delhi, New Delhi, Delhi, India; 4 Manipal Academy of Higher Education, Manipal, India; 5 Taylor's University, Subang Jaya, Selangor, Malaysia; 6 Qassim University, Buraydah, Al Qassim, Saudi Arabia

**Keywords:** economic growth, international tourist arrivals, foreign direct investment, inflation, gross domestic product, tourism

## Abstract

**Background:**

Tourism is a vital component of economic development, particularly in emerging economies like India, where international tourist arrivals contribute significantly to foreign exchange earnings, employment generation, and regional growth. While prior research has explored various determinants of tourism demand, limited empirical studies have assessed the macroeconomic underpinnings of ITA using time series models capable of handling mixed integration orders. This study investigates the short-run and long-run effects of international tourist arrivals on GDP, FDI, and inflation in India.

**Methods:**

This study uses the Autoregressive Distributed Lag bounds testing approach on annual data spanning from 1995 to 2022. The Augmented Dickey-Fuller (ADF) test was conducted to determine the stationarity properties of the time series data, after which the optimal lag structure for the ARDL model was identified using the Akaike Information Criterion (AIC). The model was built on a time series dataset spanning 1995 to 2022, and the empirical results provide both statistically significant findings and interpretive depth that are relevant to policy and theory.

**Results:**

The findings of this study confirm a statistically significant and positive relationship between GDP and international tourist arrivals in both the short and long run. The ARDL (1,2,0,2) model demonstrated strong explanatory power (Adjusted R² = 0.9565), and the bounds test confirmed the presence of cointegration among the variables. However, FDI and inflation were found to be statistically insignificant in influencing ITA. The error correction term was negative and empirically significant, indicating that approximately 51% of the disequilibrium adjusts each year toward long-run equilibrium.

**Conclusion:**

This study highlights GDP as the primary macroeconomic driver of international tourism demand in India, with implications for economic planning and tourism policy. While FDI and inflation were not significant in this model, their potential indirect effects needs further investigation.

## 1. Introduction

Tourism has progressively been known as a key element of India’s economic, sustainable and rural development. Godara and Fetrat (2022) employed econometric models to examine tourism’s role in India’s economic growth between 2000 and 2019, revealing a significant relationship between tourism revenue and GDP. Singh et al. (2023) argued that tourism industry should embrace the circular economy tenets to maintain economic value while hosting sustainability to create a resilient tourism ecosystem. Similarly, Nayak and Hanagodimath (2024) evaluated the impact of tourism on economic growth by incorporating aspects, such GDP, international tourist receipts, trade openness, and gross capital formation. Their findings suggest that trade openness and capital inflows linked to visitor spending are the major pathways through which tourism accelerates growth. Tiwari and Bisen (2023) highlighted tourism’s role in job creation, foreign exchange earnings, and regional economic convergence, particularly in underdeveloped regions. Transportation, accommodation, retail, handicraft, and local food products are just a few of the many auxiliary sectors supported by the heavily labour-intensive tourism sector. International visitors foster entrepreneurship, regional development, and job creation, often accounting for up to 10% of total employment in many countries (International Labor Organization 2024). Foreign visitor expenditure contributes significantly to foreign exchange earnings, improves current account balances, and supports capital imports. For countries with limited natural resources, tourism often serves as the main foreign exchange earner (UNWTO, ANNEX 2, 2024; Goretti et al. 2021). A study conducted by Oktriono et al. (2025) reveals a significant relationship between circular economy (CE) practices in tourism and decarbonization in the waste sector, with digitalization serving as a key enabler.

Regardless of its potential, the tourism industry faces challenges, including inadequate infrastructure, regional disparities, seasonality, degradation of environment, and limited human resource capabilities. Sustainable frameworks, green investments, improved training, and strategic planning have been recommended to safeguard that tourism development is both ecologically sustainable and economically beneficial ( Gkarane & Vassiliadis, 2024; Singhal et al., 2020; Rasool et al., 2021).

This study examines the relationship between ITA, GDP, FDI, and inflation in the context of India. The findings are expected to offer empirical evidence that can assist policymakers in formulating plans to enhance international tourism and promote broader economic growth.

The study aims to answer below research questions:

*What is the impact of India’s Gross Domestic Product (GDP), Foreign Direct Investment (FDI), and Inflation on international tourist arrivals (ITA) in the short-run and long-run?*

## 2. Literature review

With its rich cultural and natural heritage, India has enormous potential to leverage its tourism sector to achieve ambitious economic goals. A meta-analytic study covering 36 tourism-related employment studies estimated the job creation benefits of tourism expansion to range between 0.85 and 0.97, even after accounting for publication bias ( Giotis, 2024). The empirical studies testing of Tourism-Led Growth Hypothesis (TLGH) confirm tourism’s long-term contribution to economic growth. For example, a 10% increase in GDP contribution from tourism results in a 14.9% increase in long-term growth, while tourism employment, foreign visitor revenue, and capital investment also demonstrate significant elasticities ( Dritsakis, 2012; Seghir et al., 2015; Paramati et al., 2018). Pandey et al. (2024) outlined a recovery framework post-COVID-19, citing that foreign visitor arrivals in 2019 generated INR 2,109,814 crores (about USD 28.6 billion) in foreign exchange earnings, supporting more than 42 million jobs and contributing 9.2% to India’s GDP. Infrastructure spending for tourism has a ripple effect on other economic sectors, according to the socioeconomic impact assessment. India has witnessed steady growth in foreign tourist arrivals (FTAs), with 11 million arrivals in 2019, generating nearly USD 30 billion in foreign exchange revenues ( Ministry of Tourism, 2024). Government schemes, such as PRASAD and Swadesh Darshan, have supported post-pandemic recovery. States such as Kerala, Goa, Rajasthan, and Jammu & Kashmir benefit significantly from international tourism ( Press Information Bureau, 2023; Singh & Alam, 2024; Hussain Nengroo & Bhat, 2015). International tourist arrivals (ITAs) are strongly correlated with national economic performance, particularly GDP growth. Among its many facets, international tourist arrivals (ITAs) have demonstrated a strong and direct correlation with a nation’s economic success, particularly its GDP. Millions of visitors travel across borders every year, bringing with them economic opportunities and the possibility for host countries to prosper, thanks to globalization, simplified visa procedures, better transportation, and rising disposable income. Globalization, simplified visa processes, improved transportation, and rising disposable incomes have enhanced ITAs’ contributions to economic development ( Naseem & Dionísio, 2021; Enilov & Wang, 2022a; Rasool et al., 2021). Studies consistently show that a 1% increase in ITAs can result in GDP growth of 0.3% to 0.5% in South Asian and ASEAN nations ( Enilov & Wang, 2022b; Maneejuk et al., 2022).

Foreign Direct Investment (FDI) has emerged as a noteworthy factor in the growth and modernization of India’s tourism sector. Tourism, a service-intensive and capital-demanding industry, benefits extensively from external funding sources, global managerial practices, and international branding. FDI has been a crucial factor in India’s tourism modernization, enabling infrastructure growth, global management practices, and international branding ( Selvanathan et al., 2009; Sahoo & Dash, 2009; Jena et al., 2022). However, issues such as profit repatriation, the dominance of large firms, the displacement of small businesses, and environmental degradation remain concerns ( Durmanov et al., 2021; Adamu et al., 2019; Wang & Cheablam, 2025). India’s liberalized FDI policy, which allows 100% automatic investment in tourism and hospitality, continues to attract foreign investors ( Park, 2024), although inclusive policy frameworks are needed to ensure balanced and sustainable benefits ( FDI and Tourism: A Sustainable Alliance, 2024).

## 3. Data analysis and interpretation

The longitudinal dataset employed in this study was obtained from the World Bank database ( World Bank, 2025) and retrieved on 3rd August 2025. The analysis seeks to explore the relationship between international tourist arrivals, and key macroeconomic indicators, specifically gross domestic product, foreign direct investment, and inflation. To achieve this objective, the study applies the Autoregressive Distributed Lag bounds testing approach. The ARDL framework helps to estimate both short-run and long-run dynamics ( Pesaran et al., 2001). Furthermore, it accommodates regressors that are integrated of different orders, I(0) and I(1), thereby addressing a common limitation of conventional cointegration techniques which typically require all variables to be of the same integration order.

## 4. Results and discussion

The descriptive statistics shown in Table 1 presents data for four key economic indicators across 28 observations. The analysis reveals important patterns in the distribution and variability of these economic variables. Foreign Direct Investment exhibits the highest relative variability with a coefficient of variation of 50.5%. The distribution is positively skewed (0.81), indicating that most observations cluster around lower FDI levels with some countries experiencing significantly higher inflows. Despite this skewness, the Jarque-Bera test suggests the distribution approximates normality (p = 0.167). GDP per capita shows more balanced distribution characteristics with moderate skewness (0.40) and relatively high variability (CV = 38.3%). The range spans from $621 to $2,098, indicating substantial economic disparities across the sample countries. Inflation rates demonstrate significant volatility, ranging from 3.3% to 13.2% annually. The positive skewness (0.71) suggests most countries experience moderate inflation, while some face considerably higher rates. The coefficient of variation of 42.8% indicates substantial price stability differences across countries. International tourist arrivals show the highest absolute variability and the most pronounced positive skewness (0.97). Tourist numbers range dramatically from 2.1 million to 17.9 million, with a coefficient of variation of 74.2%, reflecting diverse tourism market sizes and development levels.

**Table 1.** Descriptive statistics.

Statistic	Foreign direct investment, net inflows (% of GDP)	GDP per capita (constant 2015 US$)	Inflation, consumer prices (annual %)	International tourist arrivals
Mean	1.467489	1221.436	6.640807	6941107
Median	1.499902	1120.804	6.084702	5225500
Maximum	3.620523	2098.211	13.23084	17914000
Minimum	0.472644	620.7	3.328173	2124000
Std. Dev.	0.741298	468.1163	2.843749	5153077
Skewness	0.81018	0.399012	0.713801	0.967651
Kurtosis	3.66295	1.808193	2.377089	2.476758
Jarque-Bera	3.575918	2.400121	2.830412	4.689035
Probability	0.167301	0.301176	0.242876	0.095893
Observations	28	28	28	28

Source: author’s calculations.

### 4.1 Stationarity test using ADF

To assess the order of integration of the variables, the Augmented Dickey–Fuller (ADF) test was applied, and the outcomes are presented in Table 2. The findings indicate that ITA, GDP, and FDI exhibit non-stationarity at their levels but become stationary after first differencing, implying that these variables are integrated of order one [I(1)]. Conversely, the inflation variable is stationary at its level, signifying integration at order zero [I(0)]. These results confirmed the appropriateness of the ARDL model for further analysis, as none of the variables were found to be I(2), which would violate the assumptions of the ARDL approach.

**Table 2.** Augmented Dickey -Fuller test statistic of International Tourist Arrival, GDP, FDI, and Inflation at “I(0), and I(1)”.

Variable	Null hypothesis	t - statistics	p - value	Stationary at level	Stationary at 1st difference	Order of integration
LITA	LITA has a unit root	0.528155	0.9835	No	_	I(1)
D(LITA)	D(LITA) has a unit root	-4.469229	0.0023		Yes	I(1)
LI	LI has a unit root	-4.17225	0.0041	Yes	_	I(0
LGDP	LGDP has a unit root	-0.102934	0.9395	No	_	I(1)
D(LGDP)	D(LGDP) has a unit root	-5.029253	0.0004	_	Yes	I(1)
LFDI	LFDI has a unit root	-2.069114	0.2578	No	_	I(1)
D(LFDI)	D(LFDI) has a unit root	-5.534094	0.0001	_	Yes	I(1)

Source: author’s calculations.

### 4.2 Model of the study (ARDL model specification)

The model of the study assumes that international tourist arrivals are influenced by macroeconomic indicators such as GDP, FDI, and inflation. Thus, the functional specification of the ARDL model is expressed as:

$${\text{LITA}}_{t}=f\left({\text{LITA}}_{t-1},{\text{LGDP}}_{t},{\text{LGDP}}_{t-1},{\text{LGDP}}_{t-2},{\text{LFDI}}_{t},{\mathrm{LI}}_{t},{\mathrm{LI}}_{t-1},{\mathrm{LI}}_{t-2}\right)$$

This functional relationship implies that the current value of international tourist arrivals depends on its own lag, and the current and lagged values of the selected macroeconomic variables.

**Model in Regression Form (Structural Equation)**

The functional model can be translated into the following regression form:

$${\text{LITA}}_{t}=\beta_{0}+\beta_{1}{\text{LITA}}_{t-1}+\beta_{2}{\text{LGDP}}_{t}+\beta_{3}{\text{LGDP}}_{t-1}+\beta_{4}{\text{LGDP}}_{t-2}+\beta_{5}{\text{LFDI}}_{t}+\beta_{6}{\mathrm{LI}}_{t}+\beta_{7}{\mathrm{LI}}_{t-1}+\beta_{8}{\mathrm{LI}}_{t-2}+\varepsilon_{t}$$

Where:

• LITA = Log of International Tourist Arrivals
• LGDP = Log of Gross Domestic Product
• LFDI = Log of Foreign Direct Investment
• LI = Log of Inflation
• β _0_ to β _8_ = Regression coefficients
• ε _t_ = Error term

This regression model explains how each variable, along with its lagged values, contributes to the variation in international tourist arrivals over time.

### 4.3 ARDL estimation output

**Figure 1.**
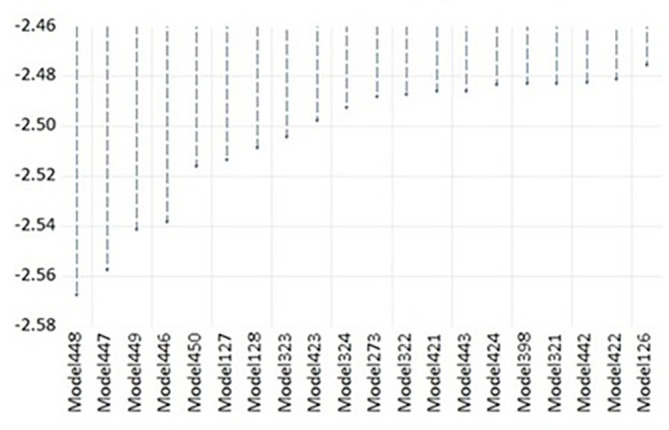
Akaike information criteria (top 20 models).

Following the stationarity analysis, the optimal ARDL model was chosen based on the Akaike Information Criterion (AIC), which identified the ARDL(1,2,0,2) model specification as the best fit. This configuration implies that one lag was included for the dependent variable (LITA), two lags for LGDP and LI, and no lag for LFDI. This model captures both short-run dynamics and long-run relationships among the variables. Table 3, presents the ARDL (1,2,0,2) estimation output. Figure 1, presents the AIC values for the top 20 models out of a total of 500 estimated models. Among all the models, Model 448, specified as ARDL(1,2,0,2), was identified as the optimal model. This selection is based on its lowest AIC value, approximately -2.56, suggesting that it provides the most efficient balance between capturing the underlying data patterns and avoiding overfitting. The substantial difference in AIC values between Model 448 and other models further reinforces its superiority in explaining the variability in the dataset with minimal information loss.

**Table 3.** ARDL (1,2,0,2) estimation output.

Variable	Coefficient	Std. error	t- statistics	p-value
LITA(-1)	0.48712	0.134887	3.61132	0.0022
LGDP	6.16984	1.147646	5.376081	0.0001
LGDP(-1)	–1.813329	1.879285	–0.964904	0.3481
LGDP(-2)	–3.494997	1.181758	–2.957456	0.0088
LFDI	0.046106	0.093885	0.491095	0.6296
LI	–0.103488	0.117041	–0.884209	0.3889
LI(-1)	–0.048318	0.126168	–0.382965	0.7065
LI(-2)	0.157609	0.111249	1.416725	0.1746
C	0.650345	0.435413	1.493626	0.1536

Source: author’s calculations.

The regression output from the ARDL (1,2,0,2) model shows a strong goodness-of-fit, (R² = 0.9704, adjusted R² = 0.9565), indicating that over 95% of the variation in international tourist arrivals could be explained by the included regressors. The Durbin-Watson statistic of 2.13 further confirmed the absence of autocorrelation, thereby reinforcing the robustness and reliability of the estimated model. Among the short-run coefficients, changes in GDP (LGDP and its lags) were found to be statistically significant, underscoring the strong influence of economic growth on international tourist arrivals in the short term. Conversely, the coefficients for FDI and inflation were statistically insignificant, suggesting their short-run effects on tourist arrivals are minimal in the present context.

The AR(1) component of the model is represented by the inclusion of the lagged dependent variable:

$${\text{LITA}}_{t}=\beta_{0}+\beta_{1}{\text{LITA}}_{t-1}+u_{t}$$

This reflects the dependence of the current level of tourist arrivals on its immediate past value, capturing momentum in tourism behavior.

The DL(2) structure in the ARDL(1,2,0,2) model is reflected in the inclusion of current and lagged values of the independent variables:

$${\text{LITA}}_{t}=\alpha +\delta_{1}{\text{LGDP}}_{t}+\delta_{2}{\text{LGDP}}_{t-1}+\delta_{3}{\text{LGDP}}_{t-2}+\delta_{4}{\text{LFDI}}_{t}+\delta_{5}{\mathrm{LI}}_{t}+\delta_{6}{\mathrm{LI}}_{t-1}+\delta_{7}{\mathrm{LI}}_{t-2}+v_{t}$$

This DL model allows the impact of independent variables to be spread over time, acknowledging that macroeconomic shocks do not affect tourism instantaneously.

### 4.4 Long-run form and bounds test

To investigate the presence of a long-run equilibrium relationship among the variables, the ARDL bounds testing procedure was employed ( Table 5). The result of F-statistic (F = 8.67) confirmed the presence of a long run cointegrating relationship among LITA, LGDP, LFDI, and LI. Table 4, presents the result of ARDL long run form test. The findings imply that, although short-run fluctuations may occur, these indicators tend to move together in the long run, maintaining a stable equilibrium relationship.

**Table 4.** ARDL long run form test.

Variable	Coefficient	Std. error	t-statistic	Prob.
LGDP	1.679759	0.215855	7.781883	0
LFDI	0.089897	0.17799	0.505062	0.6193
LI	0.011313	0.166248	0.068052	0.9465
C	1.268206	0.706818	1.794011	0.0906

EC = LITA - (1.6798LGDP + 0.0899LFDI + 0.0113*LI + 1.2680), Source: author’s calculations.

**Table 5.** Bounds test.

Test statistic	Value	Level	I(0)	I(1)
	Asymptotic : n = 1000			
F - statistic k	8.6669064	10%	2.37	3.2
		5%	2.79	3.67
		2.50%	3.15	4.08
		1%	3.65	4.66

Source: author’s calculations.

**Table 6.** ECM form estimation.

Variable	Coefficient	Std. error	t-statistic	p-value
D(LGDP)	6.1698	0.7995	7.7174	0
D(LGDP(-1))	3.495	1.0042	3.4805	0.0029
D(LI)	-0.1035	0.0823	-1.2581	0.2254
D(LI(-1))	-0.1576	0.0902	-1.7467	0.0987
Cointeq(-1)*	-0.5129	0.0701	-7.3174	0

Source: author’s calculations.

The long-run coefficients revealed that GDP had a statistically significant and positive relationship with international tourist arrivals. Specifically, a 1% increase in GDP was associated with an approximate 1.68% increase in international tourist arrivals, with a p-value less than 0.01( Table 3). The results aligns with the existing literature findings, suggesting that higher GDP levels enhance a country’s ability to attract tourists by improving infrastructure, accessibility, and service quality ( Song & Li, 2008). In contrast, the long-run coefficients for LFDI and LI were statistically insignificant, indicating that, in the context of this study, foreign investment and inflationary trends do not have a substantial long-term effect on inbound tourism. The constant term was marginally significant, possibly indicating a base level of tourist arrivals independent of macroeconomic conditions.

### 4.5 Short run estimation

Table 6 presents the results of the ECM estimation. The error correction coefficient was -0.5129 and is statistically significant (p < 0.05).This finding suggests that any short-term disequilibrium between the variables does not persist indefinitely but rather converges back toward the long-run equilibrium path at a relatively fast pace. Specifically, about 51% of the deviations from the equilibrium level are corrected within a single period, suggesting that the model has a strong self-adjusting tendency. Within the short-run dynamics, both the contemporaneous and lagged first differences of GDP (D(LGDP) and D(LGDP(–1))) were found to be statistically significant. In contrast, inflation and its lagged values remained insignificant, suggesting that their short-run impact on tourist arrivals is minimal.

Overall, the results from all steps of the ARDL analysis point toward GDP being the most influential macroeconomic factor driving international tourist arrivals in India. The significance of the error correction coefficient confirms the existence and stability of the long-run relationship, while FDI and inflation, though theoretically relevant, do not demonstrate statistically significant impacts within this empirical framework.

### 4.6 Heteroskedasticity test

To examine the assumption of homoskedasticity in the residuals of the ARDL (1, 2, 0, 2) model, the Breusch–Pagan–Godfrey test was employed (F-statistic = 0.3458 p-value = 0.9349, and Chi-square statistics = 0.8822, p-value = 0.9351). The results indicate that the residuals exhibit homoscedasticity, thereby providing no evidence of heteroskedasticity within the sample. The outcome validates one of the key classical linear regression assumptions, reinforcing the reliability of the estimated standard errors and the strength of inference drawn from the ARDL model in modelling the macroeconomic determinants of international tourist arrivals in India.

### 4.7 Serial correlation test

To validate the assumption of residual independence, the Breusch–Godfrey LM test for serial correlation was performed. The results yielded an F-statistic of 1.387 (p = 0.2801) and a Chi-square statistic of 4.0579 (p = 0.1315), both of which are statistically insignificant at the 5% level. These findings indicate that the null hypothesis of no serial correlation in the residuals up to the second lag cannot be rejected. Thus, the model passes the serial correlation diagnostic, confirming that the residuals are not autocorrelated and are independently distributed. This enhances the reliability of the coefficient estimates and ensures the robustness of inference drawn from the ARDL model in the context of analysing macroeconomic determinants of international tourist arrivals in India.

### 4.8 Residual diagnostics and model adequacy

**Figure 2.**
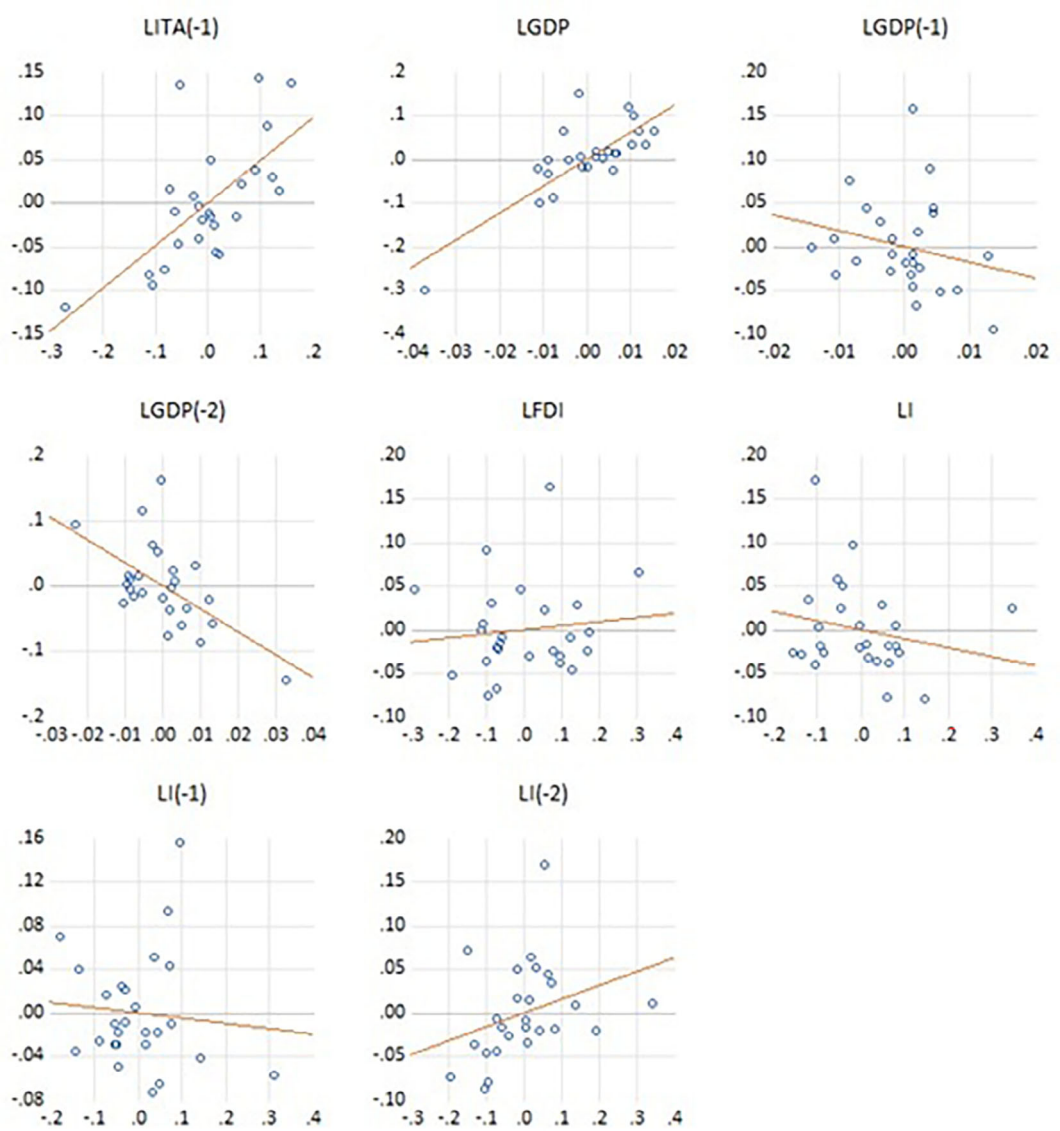
Residuals vs. variables (partialled on gradients).

The residuals-versus-variables diagnostic plots presented in Figure 2 provide an assessment of the adequacy of the ARDL model specification. The scatterplots illustrate the distribution of residuals against each explanatory variable and their respective lagged terms, with fitted trend lines superimposed to detect any systematic patterns. The residuals appear to be randomly dispersed around the zero line across most variables, indicating that the assumptions of linearity and homoscedasticity are broadly satisfied. Notably, the plots for lagged international tourist arrivals [LITA(-1)] and GDP (LGDP) display stronger linear patterns, suggesting their significant explanatory power in the model. By contrast, the residual distributions for FDI (LFDI) and lagged inflation terms [LI(-1), LI(-2)] reveal weaker or more scattered associations, reflecting a relatively limited direct impact of these variables. Importantly, the absence of pronounced curvature or heteroscedastic spread in the residuals reinforces the structural adequacy and reliability of the estimated ARDL model, lending further credibility to the subsequent long-run and short-run estimates.

### 4.9 Jarque–Bera normality test

To assess the assumption of normality of the residuals in the estimated ARDL (1,2,0,2) model, the Jarque–Bera (JB) test was conducted. The Jarque–Bera (JB) statistic (JB = 11.17, p = 0.0038), at the 5% significance level, shows that the null hypothesis of normally distributed residuals is rejected, thereby indicating evidence of non-normality in the error terms. Despite this, the deviation from normality is not severe and can be attributed to the relatively small sample size (n = 26). Moreover, as the model satisfies other diagnostic conditions such as stability (as confirmed by the CUSUM test) and no serial correlation, the impact of non-normal residuals on estimation and inference is likely minimal. Nevertheless, this observation should be acknowledged as a limitation when interpreting hypothesis testing results derived from the ARDL model.

### 4.10 CUSUM stability test

**Figure 3.**
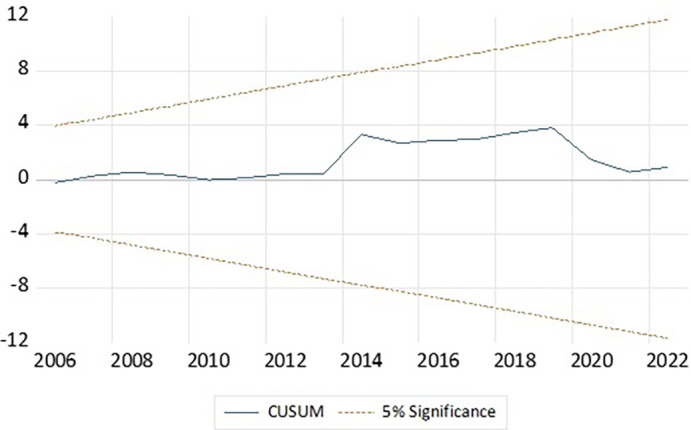
Cusum test. Source: author’s calculations.

To assess the structural stability of the estimated ARDL(1,2,0,2) model, the CUSUM test was conducted. Figure 3 presents the CUSUM plot, where the test statistic (blue line) consistently remains within the 5% significance boundaries (orange dashed lines) over the entire sample period from 1995 to 2022, thereby indicating the structural stability of the model, confirming the relationship between ITA and the macroeconomic indicators - GDP, FDI, and inflation remained stable during the observed period. The stability of the model further enhances the credibility and reliability of the long-run and short-run coefficients estimated through the ARDL framework. This robustness supports the use of the model for policy interpretation and forecasting within the scope of India’s inbound tourism dynamics.

### 4.11 Ramsey RESET test

To evaluate the adequacy of functional form of the ARDL(1,2,0,2) model, Ramsey RESET test was conducted. It included square of the fitted values as an additional regressor to detect potential specification errors. The results showed an F-statistic of 3.4271 with a p-value of 0.0827, which is not statistically important at the conventional 5% level but indicates marginal significance at the 10% level. However, the Likelihood Ratio (LR) test yielded a statistic of 5.0461 with a p-value of 0.0247, which is significant at the 5% threshold. This mixed outcome proposes some evidence of possible functional form misspecification **,** particularly from the LR perspective. While the RESET test does not identify the exact source of the misspecification, it signals that non-linear terms or omitted variables may be contributing to model inefficiency. However, model satisfies other key diagnostic checks such as the non-appearance of serial correlation, homoskedasticity, and structural stability results of the RESET test should be viewed as a prompt for further model refinement rather than a rejection of model validity.

## 5. Discussion and conclusion

The findings of the study shows that there is consistently strong and positive relationship between GDP and international tourist arrivals, both in the short run and long run. This suggests that as the Indian economy grows, its ability to attract international tourists improves. This relationship can be attributed to several economic and behavioral mechanisms. First, GDP growth often leads to increased government investment in infrastructure, transportation networks, urban development, and tourism-specific amenities all of which enhance the appeal of the destination ( Brida & Risso, 2010). Second, economic prosperity typically coincides with improved global perceptions of safety, stability, and service quality, further boosting a country’s tourism competitiveness ( Crouch & Ritchie, 1999).

The positive long-run coefficient of GDP (1.6798) indicates a substantial elasticity of tourist arrivals with respect to economic performance. This aligns with the demand-side tourism theory, which suggests that macroeconomic indicators, especially income and output levels, are foundational determinants of tourism flows ( Song & Li, 2008). The short-run results further reinforce this pattern: current and lagged changes in GDP significantly predict variations in tourist arrivals. This dynamic relationship suggests that tourism demand is responsive not only to long-term trends in economic performance but also to more immediate economic signals, such as quarterly or annual growth rates.

However, the effects of FDI and inflation on international tourist arrivals for both short- and long-run specifications were found statistically insignificant. This outcome, while perhaps unexpected, is consistent with some previous studies in the literature, particularly in large and diversified economies where FDI is often dispersed across multiple sectors not directly linked to tourism. The insignificance of FDI could indicate that foreign investment, unless specifically directed toward tourism-supporting infrastructure (e.g., hospitality, transportation, attractions), does not automatically translate into increased tourist arrivals. Likewise, inflation’s negligible influence might be explained by India’s relative affordability as a tourism destination. Even with moderate inflationary trends, India remains cost-competitive compared to many Western and Asian countries, thereby softening the price sensitivity of inbound travellers ( Tang, 2011).

Moreover, the importance of error correction term in model highlights the long-run equilibrium among the indicators. This finding adds support to the economic equilibrium theory in tourism modelling, which posits that tourism markets tend to correct themselves and return to equilibrium over time after external or internal disturbances ( Song et al., 2012).

The overall robustness of the ARDL model, demonstrated by a high adjusted R² (0.9565), a significant F-statistic (p < 0.01), and a Durbin-Watson statistic near 2 (indicating no autocorrelation), confirms the model’s reliability in capturing the sturdy tourism-macroeconomy relationship. These statistical strengths, combined with the theoretical relevance of the findings, enhance the credibility and policy usefulness of the study.

In conclusion, this research establishes that GDP is a critical macroeconomic determinant of international tourist arrivals to India. The influence of FDI and inflation, although theoretically plausible, appears empirically weak in this model. This could reflect the need for more targeted sectoral investment and price control mechanisms within the tourism ecosystem. The findings reinforce the importance of macroeconomic stability and growth as cornerstones of sustainable tourism development. For India, a country with immense tourism potential, aligning tourism policies with broader economic planning will be essential in optimizing inbound travel flows and achieving long-term sectoral growth.

## 6. Practical implications

The findings of this study offer significant practical value for policymakers, tourism boards, economic planners, and hospitality industry stakeholders in India and other developing economies. A growing economy not only reflects consumer and investor confidence but also allows governments to allocate greater resources for infrastructure development, safety, digitalization, and destination marketing—all of which contribute to a positive visitor experience ( Gooroochurn & Sugiyarto, 2005).

Policymakers can use this insight to design integrated tourism strategies that are synchronized with national economic development plans. For example, tourism investments can be targeted in regions experiencing economic growth to reinforce a cycle of reciprocal benefits, where tourism supports local economies and economic development improves tourism services and infrastructure. Similarly, government stimulus packages or tax benefits during economic downturns can help buffer the tourism sector, which is highly sensitive to economic shocks.

Furthermore, while FDI did not show statistically significant results in the current model, the potential long-term benefits of targeted foreign investment in tourism-supporting sectors such as hospitality, transport, and smart tourism infrastructure should not be dismissed. Governments may consider offering investment incentives, public–private partnership (PPP) frameworks, and policy clarity to attract high-quality foreign capital into the tourism and hospitality industry. It aligns with global trends where nations like the UAE, Vietnam, and Thailand have leveraged FDI to enhance their tourism offerings ( UNCTAD, 2022).

Although inflation appeared statistically insignificant, its practical implications should be interpreted with caution. Persistent inflation could erode service quality, raise travel costs, and reduce tourism’s price competitiveness in the long term ( Crouch, 1994). Hence, maintaining price stability through sound monetary policy is essential to protect the affordability of tourism services and safeguard visitor perceptions of value for money.

In addition, tourism practitioners - hoteliers, travel agents, event planners, and destination managers can benefit from macroeconomic monitoring to anticipate demand fluctuations. For instance, in periods of strong GDP growth, businesses may prepare for increased tourist flow by expanding offerings, hiring seasonal staff, or investing in service quality enhancements. Likewise, during economic downturns, adaptive pricing strategies and domestic tourism promotion could help mitigate potential losses.

## 7. Theoretical implications

The model confirms that macroeconomic fundamentals, particularly GDP, play a foundational role in explaining tourism demand over both the short and long run. This reinforces demand-side theories of tourism that posit economic development as a key determinant of travel behavior and destination competitiveness ( Lim, 1997; Crouch, 1995).

By validating the impact of GDP on international tourist arrivals, the study empirically supports the Tourism-Led Growth Hypothesis (TLGH), which suggests a bi-directional and reinforcing relationship between tourism and economic growth. However, in this study, it is GDP that drives tourism arrivals, indicating the dominance of the economy-led tourism growth model ( Oh, 2005). This distinction is particularly relevant for emerging economies like India, where macroeconomic performance influences both government spending on tourism infrastructure and consumer sentiment among foreign travellers.

The absence of statistically significant effects of FDI and inflation also invites theoretical reflection. While many studies assume that macroeconomic stability (e.g., low inflation) and capital inflows (e.g., FDI) automatically enhance tourism competitiveness, this research suggests that such relationships may be indirect, contingent on sectoral targeting, institutional quality, or lagged effects not captured within a 26-year annual data window. This supports a more nuanced view of the macroeconomic tourism nexus, highlighting the need for theoretical models that consider mediating and moderating variables such as governance, infrastructure utilization, exchange rate regimes, or geopolitical risk.

Methodologically, the application of the ARDL approach strengthens the case for using flexible models in tourism demand forecasting, particularly when variables are integrated at mixed orders. The model’s ability to differentiate between short-run fluctuations and long-run equilibrium adjustments provides richer insights than static regression models. Additionally, the importance of the error correction term adds support to the dynamic adjustment theory, which argues that tourism flows gradually return to equilibrium after economic disturbances ( Song et al., 2012).

Finally, by focusing on India, which is a large, diverse, and increasingly significant player in global tourism, this study adds to the geographically diversified empirical base of tourism economics. Most tourism demand modelling studies have focused on developed economies or small island destinations; thus, this research fills a critical gap by contextualizing the findings within a populous, developing nation facing unique socio-political and infrastructural challenges and opportunities.

## 8. Research limitations

Despite the valuable findings, the study is subject to several limitations. First, the analysis is based on aggregate annual data for India, which may mask regional disparities in tourism dynamics and economic conditions. Future studies could benefit from using disaggregated (state-level or destination-specific) data to capture regional variations more precisely.

Second, the model includes only three macroeconomic variables. Although GDP, FDI, and inflation are widely studied, other potentially relevant factors such as exchange rates, political stability, visa policies, environmental sustainability, or pandemic-related variables (e.g., COVID-19 disruptions) were not incorporated. Including such variables could offer a more comprehensive understanding of tourism demand drivers ( Goh, 2008).

Third, the ARDL model, while robust for short samples and mixed integration orders, does not account for potential structural breaks or non-linear relationships. Future research could consider using threshold models, Markov-switching models, or vector error correction models (VECM) to capture possible regime shifts or non-linearities in the tourism-macroeconomy relationship.

Lastly, this study is specific to India. To enhance external validity, comparative studies across other emerging markets or tourism-dependent economies could provide richer insights into the global applicability of these findings.

## Ethics and consent statement

This study did not involve human participants, animals, or any sensitive personal data. Consequently, no formal ethical approval or informed consent was required. The research was conducted entirely using publicly available secondary data obtained from official sources, ensuring that all analyses adhered to standard academic and research integrity guidelines. The authors confirm that the study complies with ethical principles related to transparency, reproducibility, and responsible use of data.

## Data Availability

The data employed in this study are publicly accessible through the World Bank Open Data platform ( https://data.worldbank.org), and also openly available on Zenodo at 10.5281/zenodo.17088197 ( Singh A., 2025). Zenodo. Secondary Data extracted from world bank: https://www.worldbank.org/ext/en/home ( Singh, 2025). This project contains the following underlying data:
• World bank data set xlsx Data are available under the terms of the Creative Commons Attribution 4.0 International license (CC-BY 4.0) https://creativecommons.org/licenses/by/4.0/.
