# Supplementary material for: Assessing the Macroeconomic Determinants of International Tourist Arrival in India: An ARDL Bounds Testing Approach
Source: F1000Res. 2025 Nov 5;14:1215. [Version 1] doi: 10.12688/f1000research.170280.1 (PMC12658354; doi:10.12688/f1000research.170280.1)
Supplement: Peer review history

# Reviewer report: Reviewer response for version 1

**DOI:** 10.5256/f1000research.187722.r431252

**Published:** 2025-11-26

**Authors:** Dr. Aruditya Jasrotia

**Affiliations:** 1 Amity University, Noida, India

The research paper titled **“Assessing the Macroeconomic Determinants of International Tourist Arrival in India: An ARDL Bounds Testing Approach”** presents a relevant and timely analysis of tourism demand within a macroeconomic framework. The study is well-structured and employs a rigorous ARDL bounds testing methodology appropriate for handling variables of mixed integration orders. One of its core strengths lies in the clear identification of GDP as a statistically significant long-run and short-run determinant of international tourist arrivals, supporting the tourism–growth nexus in the Indian context. However, the abstract contains directional ambiguity, at times implying that tourist arrivals influence GDP rather than GDP influencing arrivals, which should be clarified for conceptual consistency. While the use of annual data from 1995 to 2022 strengthens the reliability of the study, the paper would benefit from the latest statistics. The finding that FDI and inflation are insignificant offers meaningful insight but requires deeper theoretical justification, especially since previous studies have shown mixed evidence regarding their impact on tourism demand. The conclusion appropriately highlights policy relevance, though it could be strengthened through concrete recommendations linking tourism planning with macroeconomic management. Overall, the study offers valuable empirical evidence, but enhancing theoretical grounding, expanding discussion of insignificant variables, and providing stronger justification for variable selection would improve its academic robustness and publication readiness.

Is the work clearly and accurately presented and does it cite the current literature?

Yes

If applicable, is the statistical analysis and its interpretation appropriate?

Yes

Are all the source data underlying the results available to ensure full reproducibility?

Yes

Is the study design appropriate and is the work technically sound?

Yes

Are the conclusions drawn adequately supported by the results?

Partly

Are sufficient details of methods and analysis provided to allow replication by others?

Yes

Reviewer Expertise:

Tourism Management

I confirm that I have read this submission and believe that I have an appropriate level of expertise to confirm that it is of an acceptable scientific standard.

# Reviewer report: Reviewer response for version 1

**DOI:** 10.5256/f1000research.187722.r431253

**Published:** 2025-11-24

**Authors:** Dr. Kriti Priya Gupta

**Affiliations:** 1 Symbiosis University, Pune, Maharashtra, India

Abstract: The abstract uses acronyms such as **ITA**, **GDP**, **FDI**, and **ARDL** without defining them at first mention. In the abstract, the authors state that the study “investigates the short-run and long-run effects of *international tourist arrivals on* GDP, FDI, and inflation,” which contradicts the functional form used in the study: LITAt=f(LITAt−1,LGDPt,LGDPt−1,LGDPt−2,LFDIt,LIt,LIt−1,LIt−2). The abstract must be corrected to reflect that GDP, FDI, and inflation influence ITA, not the other way around.

 Introduction: 

 1) The introduction reports prior studies individually but does not synthesize them to highlight patterns, contradictions, or gaps in the literature.The authors must clearly state:

- Why this study is needed.
- What gaps exist in existing tourism demand research (e.g., limited macroeconomic determinants tested for India, lack of dynamic time-series models, mixed integration orders).
- How the study contributes to theory or policy.
- The origin of the research problem—why macroeconomic determinants of tourism arrivals are important specifically for India.

2) The sentence:

*“Similarly, Nayak and Hanagodimath (2024) evaluated the impact of tourism on economic growth by incorporating aspects, such GDP, international tourist receipts, trade openness, and gross capital formation…” *contains grammatical errors.

 3) The manuscript states:

*“Regardless of its potential, the tourism industry faces challenges, including inadequate infrastructure, regional disparities, seasonality, degradation of environment, and limited human resource capabilities.” *These claims require appropriate citations from credible sources.

Is the work clearly and accurately presented and does it cite the current literature?

Partly

If applicable, is the statistical analysis and its interpretation appropriate?

Yes

Are all the source data underlying the results available to ensure full reproducibility?

Yes

Is the study design appropriate and is the work technically sound?

Yes

Are the conclusions drawn adequately supported by the results?

Yes

Are sufficient details of methods and analysis provided to allow replication by others?

Yes

Reviewer Expertise:

Behavioural aspects of technology adoption

I confirm that I have read this submission and believe that I have an appropriate level of expertise to confirm that it is of an acceptable scientific standard, however I have significant reservations, as outlined above.

# Reviewer report: Reviewer response for version 1

**DOI:** 10.5256/f1000research.187722.r431248

**Published:** 2025-11-21

**Authors:** Rahul Pratap Singh Kaurav

**Affiliations:** 1 Fore School of Management, New Delhi, Delhi, India

Dear Authors,

 Using the ARDL bounds testing method, this study provides an organised and empirically sound investigation of the macroeconomic factors impacting foreign visitor arrivals in India. The work is notable for its thorough diagnostics, rigorous methodology, and strong differentiation between short-run and long-run dynamics. The authors offer a sophisticated explanation of how macroeconomic stability propels tourist growth by skilfully combining GDP, FDI, inflation, and exchange rate determinants. The economic-led tourist expansion hypothesis in the Indian context is reinforced by the solid empirical results, which are backed by a solid theoretical foundation and comprehensive literature contextualisation.

 By providing practical insights into how economic factors influence inbound tourism demand, the work significantly advances tourism economics and policy research. The results are important for academics as well as policymakers who want to match macroeconomic goals with tourism development. This study is a commendable and indexable piece of research because of its logical organisation, clear presentation, and useful policy implications.

Is the work clearly and accurately presented and does it cite the current literature?

Yes

If applicable, is the statistical analysis and its interpretation appropriate?

Yes

Are all the source data underlying the results available to ensure full reproducibility?

Yes

Is the study design appropriate and is the work technically sound?

Yes

Are the conclusions drawn adequately supported by the results?

Yes

Are sufficient details of methods and analysis provided to allow replication by others?

Yes

Reviewer Expertise:

marketing, tourism, destination

I confirm that I have read this submission and believe that I have an appropriate level of expertise to confirm that it is of an acceptable scientific standard.
